# Identification of claudin-4 as a marker highly overexpressed in both primary and metastatic prostate cancer

**DOI:** 10.1038/sj.bjc.6604486

**Published:** 2008-07-22

**Authors:** K A Landers, H Samaratunga, L Teng, M Buck, M J Burger, B Scells, M F Lavin, R A Gardiner

**Affiliations:** 1 Department of Surgery, University of Queensland, Herston, Qld, Australia; 2 Queensland Institute of Medical Research, Herston, Qld, Australia; 3 Sullivan Nicolaides Pathology, Taringa, Qld, Australia; 4 Department of Urology, Royal Brisbane and Women's Hospital, Herston, Qld, Australia

**Keywords:** prostate cancer, marker expression, RNA and protein, claudin-4

## Abstract

In the quest for markers of expression and progression for prostate cancer (PCa), the majority of studies have focussed on molecular data exclusively from primary tumours. Although expression in metastases is inferred, a lack of correlation with secondary tumours potentially limits their applicability diagnostically and therapeutically. Molecular targets were identified by examining expression profiles of prostate cell lines using cDNA microarrays. Those genes identified were verified on PCa cell lines and tumour samples from both primary and secondary tumours using real-time RT–PCR, western blotting and immunohistochemistry. Claudin-4, coding for an integral membrane cell-junction protein, was the most significantly (*P*<0.00001) upregulated marker in both primary and metastatic tumour specimens compared with benign prostatic hyperplasia at both RNA and protein levels. In primary tumours, claudin-4 was more highly expressed in lower grade (Gleason 6) lesions than in higher grade (Gleason ⩾7) cancers. Expression was prominent throughout metastases from a variety of secondary sites in fresh-frozen and formalin-fixed specimens from both androgen-intact and androgen-suppressed patients. As a result of its prominent expression in both primary and secondary PCas, together with its established role as a receptor for *Clostridium perfringens* enterotoxin, claudin-4 may be useful as a potential marker and therapeutic target for PCa metastases.

The prostate-specific antigen (PSA) and transrectal ultrasound biopsy approach for detection of prostate cancer (PCa) has resulted in a stage shift in terms of earlier diagnosis (Hankey *et al*, 1999). However, even when the tumour is considered localised clinically, a significant proportion of patients already have micrometastases, which declare themselves subsequent to treatment with curative intent (Freedland *et al*, 2003). Currently, localised tumour is potentially curable but metastatic cancer is not (Fusi *et al*, 2004). Intuitively, understanding key molecular events leading to PCa will lead to the identification of potential diagnostic and/or prognostic biomarkers, more reliable and earlier detection together with implications for diagnostic and therapeutic targeting (Hanahan and Weinberg, 2000). Methods used for the identification of potential markers for PCa include gene expression analysis (e.g. microarrays and quantitative PCR) and protein markers (e.g. histopathology, mass spectrometry) (Ornstein and Tyson, 2006). Numerous genes that are up- or downregulated in PCa have been identified with *α*-methylacyl-CoA racemase (*AMACR)* (Rubin *et al*, 2002), prostate-specific membrane antigen (*PSMA)* (Burger *et al*, 2002), *Hepsin* (Stephan *et al*, 2004), *DD3/PCA3* (Hessels *et al*, 2003) and *hTERT* (Sommerfeld *et al*, 1996) among the more common (Ross *et al*, 2003).

In PCa detection, greater value would be afforded if markers were not only able to distinguish between benign and malignant tumours, but also to indicate innate tumour aggressiveness (including likelihood of metastasis). Their value would be enhanced even further if expression in primary tumours correlated with those in metastases and most valuable if they were also targets for imaging and therapeutic strategies. Evidence to date indicates that provision of such information is beyond the scope of any one marker (Landers *et al*, 2005), and it is apparent that different genes have relative strengths and weaknesses in this context. Thus, this study aimed to correlate the use of potential biomarkers of PCa primary lesions with metastases by comparing expression profiles of normal prostate cells with those of clinically significant PCa cells from primary and from metastatic tumours.

## Materials and methods

### Tissue culture

RWPE1 cells, derived from the peripheral zone of a histologically normal adult human prostate and transformed with a single copy of the human papilloma virus-18 (HPV-18), and metastatic PCa cell lines ALVA41, DU145, LNCaP and PC3 were obtained from Professor Judith Clements (Queensland University of Technology, Australia). The PCa cell lines were cultured in 10% (v/v) FCS in RPMI-1640 medium with 100 U ml^−1^ penicillin-G (GIBCO™ Invitrogen Corporation MT Waverley, Australia) and 100 U ml^−1^ streptomycin (GIBCO); RWPE1 cells were cultured in keratinocyte serum-free media (KSFM; GIBCO) supplemented with 5 ng ml^−1^ human recombinant epidermal growth factor and 0.05 mg ml^−1^ bovine pituitary extract. Cell cultures were maintained at 37°C in a humidified atmosphere of 5% CO_2_.

### Specimen collection

All tissue specimens collected were obtained from consenting patients Royal Brisbane Women's Hospital, Queensland, as approved by the Institutional Ethics Committee. Primary PCa tissue specimens were obtained from patients undergoing either a radical prostatectomy (RP) or a transurethral resection of prostate (TURP) with secondary tumours provided by HS from both androgen-intact and androgen-suppressed patients. Benign prostatic hyperplasia (BPH) tissue specimens were obtained from men who had either TURP or an open enucleative prostatectomy. Tissue fragments were frozen immediately using liquid nitrogen and transported on dry ice for storage at −70°C with closely adjacent tissue specimens placed in OCT and snap frozen or formalin-fixed and paraffin-embedded. Tissues prepared for histology were examined to confirm the diagnosis of BPH or PCa and to determine the proportion of epithelial cells to stromal cells. Nonsampled tissues were analysed histopathologically, as per routine practice, to confirm the clinical diagnosis and, for PCa, to determine Gleason scores and the percentage of tumour cells present. Additional paraffin sections with high-grade prostatic intraepithelial neoplasia (HG-PIN), PCa or metastatic tissue were obtained by HS. Control benign sections, harvested from deceased individuals following appropriate consent, were kindly provided by Associate Professor David Horsfall (Hanson Institute, Adelaide).

### RNA extraction

Tissue fragments were homogenised using a Polytron PK® homogeniser, in 1 ml TRI Reagent® (Sigma-Aldrich, Castle Hill, New South Wales, Australia) per 50 mg of tissue. Prostate cell lines were grown until confluent and 5–10 × 10^6^ cells collected, washed in PBS and resuspended in TRI Reagent and subsequently extracted using the recommended protocol.

### cDNA microarrays

Twenty micrograms of total RNA from a prostate cell line and the reference RNA, isolated as described above, were indirectly labelled with aminoallyl-dUTP (Amersham Biosciences, Piscataway, NJ, USA) by reverse transcription using an oligo(dT)_15_ primer (Roche Diagnostics, Castle Hill, New South Wales, Australia) and Superscript III™ (Invitrogen). Following synthesis, aminoallyl-labelled cDNA was purified, dried and the pellet resuspended in 100 mM sodium carbonate, pH 9.0. Cy5 or Cy3 dyes (Amersham Biosciences) were added to respective samples and the coupling reaction allowed to proceed for 1 h. Unincorporated dyes were removed from the reactions with 4 M hydroxylamine. The Cy3- and Cy5-labelled cDNAs were combined and purified using QIAquick™ PCR kit (Qiagen, Doncaster, Victoria, Australia). Human Cot1 DNA (10 *μ*g) and polydA (2 *μ*g) were added to the purified probe mix and dried before redissolving in 4 × SSC, 50% deionised formamide and 0.25% SDS. Following incubation at 95°C for 5 min and 45°C for 90 min, the labelled probe mix was loaded on to a Human V6 custom microarray prepared in house. Following hybridisation, the microarray chip was washed in 0.2 × SSC, 0.05% SDS, followed by 0.2 × SSC and centrifuged at low speed to dry. Cy3 and Cy5 fluorescence on the microarray chip were detected with a GMS 418 Array Scanner (Genetic Microsystems, Woburn, MA, USA) using Imagene™ (BioDiscovery, El Segundo, CA, USA). Data generated using Imagene were normalised and filtered using the GeneSpring™ (GeneWorks Pty Ltd, The Barton, South Australia) software. Normalisations included per spot and per chip intensity-dependent (Lowess) normalisations; data transformation set measurements less than 0.01–0.01 and per chip normalisations to the 50th percentile. The Human V6 microarray, consisting of 4600 cDNA probes ∼200 bp in length spotted in duplicate on a single chip, was developed in-house and its use has been described by us previously (Burger *et al*, 2002).

### Quantitative reverse transcription PCR

Primers for the candidate genes were designed using Taqman (PE Applied Biosystems, Foster City, CA, USA) guidelines and optimised using Amplitaq Gold. The following primers were synthesised: *β*2-microglobulin forward 5′-TGAATTCGTATGTGTCTGGGT-3′, *β*2-microglobulin reverse 5′-CCTCCATGATGCTGCTTACAT-3′; claudin-4 forward 5′-AGCTCTGTGGCCTCAGGACTCT-3′, claudin-4 reverse 5′-CAGTGATGAATAGCTCTTCTTAAATTACAA-3′; DAD1 forward 5′-CAACCCACAGAACAAAGCGG-3′, DAD1 reverse 5′-CTGCCATCTCCAGAACTCTTATCC-3′; POX1 forward 5′-GCTGGAAACCTGGCAGTGATAC-3′, POX1 reverse 5′-CAAAGGAAGAAAGGCTGGTCTCTC-3′; TPD52 forward 5′-GCTGCTTTTTGCTCTGTTGGC-3′ and TPD52 reverse 5′-TTTTCTGGAAGAGGCTCCGTGG-3′. Real-time PCRs were performed in 15 *μ*l volumes (cDNA, 1 × Plantinum® SYBR Green Quantitative PCR SuperMix-UDG (Life Technologies™) and 5 pmol each primer) using a 32-well Rotorgene real-time PCR machine (Corbett Life Science, Mortlake, New South Wales, Australia). Cycles consisted of a 94°C, 2 min hot start, 15 s 94°C denaturing step, a 15 s 55°C annealing step and a 15 s 72°C extension step for 35–40 cycles. The PCR finished with a melt curve between 55 and 100°C. Expression of each gene was calculated using a standard curve determined by expression of *β*2-microglobulin (*β2M*) in the control normal prostate cell line, RWPE1. Prostate-specific antigen was used to confirm that the RNA was of prostatic origin. Expression of each gene was determined in an average of 18 BPH, 17 PCa and 5 metastatic lesions. The transcript value of each gene was normalised in Excel by determining ratio of the candidate gene transcript/*β2M* transcript value. Results are presented as mean±s.e.

### Western blot analysis

Cells were washed and lysed in lysis buffer (20 mM Tris pH 8.0, 1 mM EDTA, 1 mM (*p*-amidinophenyl) methanesulfonyl fluoride hydrochloride and 1% (v/v) Triton X-100) as described by Liu *et al* (1997). Protein concentration was estimated using the DC protein Assay (Bio-Rad Laboratories Pty Ltd, Gladesville, New South Wales, Australia) and BSA protein standards. The protein (20 *μ*g) samples were mixed with loading buffer (2 × : 0.5 ml *β*-mercaptoethanol, 20% (v/v) glycerol, 2% (v/v) SDS, 37.3 M bromophenol blue, 0.25 M Tris, pH 8.8) and heated to 100°C for 2 min. Samples were resolved on a 15% polyacrylamide gel (15% (v/v) acrylamide, 0.375 M Tris pH 8.8, 0.1% (v/v) SDS, 0.1% (w/v) ammonium persulfate and 4 *μ*l TEMED in 10 ml) with stacking gel (1.5% (v/v) acrylamide, 0.038 M Tris pH 6.8, 0.03% (v/v) SDS, 0.03% (w/v) ammonium persulfate and 3 *μ*l TEMED) at 20 mA in running buffer (25 mM Tris, 0.19 M Glycerine, 0.1% (v/v) SDS in H_2_O). Proteins were transferred onto a nitrocellulose membrane in a carbonate buffer (10 mM NaHCO_3_, 3 mM Na_2_CO_3_, 20% (v/v) methanol in H_2_O) at 40 V for 3 h at 4°C. Membranes were blocked in 5% (w/v) skim milk powder in 0.05% (v/v) Tween 20 in PBS (PBST) for 1 h at RT and probed overnight with anti-claudin-4 mouse antibody (3 *μ*g per 10 ml in PBST; Zymed® Laboratories, San Francisco, CA, USA). Secondary anti-mouse IgG antibody conjugated to horseradish peroxidase (HRP) (Chemicon Australia Pty Ltd, Boronia, Victoria, Australia) was added for 1 h at 4°C. Following washing with PBST, proteins were detected using ECL Western blotting procedure (Amersham/GE Healthcare Biosciences Pty Ltd, Rydalmere, New South Wales, Australia) following the manufacturer's instructions. *β*-actin was also assayed to confirm protein loading was equal for all samples.

### Immunofluorescent staining of claudin-4 in prostate cell lines

ALVA41, DU145, LNCaP, PC3, RWPE1 and HeLa cells (1 × 10^4^) cultured in six-well plates were washed in 1% (v/v) FCS in PBS and fixed in 4% paraformaldehyde for 30 min at room temperature. Cells were washed, permeabilised with 0.1% (v/v) Triton X-100 in PBS for 30 min, washed again and blocked in 5% skim milk in PBST for 30 min. After removal of blocking reagent, the fixed cells were washed and incubated with the mouse anti-claudin-4 antibody (3 *μ*g ml^−1^) for 1 h at RT. Cells were again washed and incubated with the secondary anti-mouse IgG conjugated to FITC for 20 min. Cells were rinsed, stained with DAPI for 10 min, washed again and the coverslip inverted onto a slide with a drop of 80% (v/v) glycerol. The cells were visualised using a Carl Zeiss fluorescence microscope (Axioskop 2 plus MOT).

### Immunohistochemistry on paraffin-embedded sections

The paraffin-embedded prostate tissue sections were obtained from patients with a range of prostate conditions, including 29 benign prostate, 19 BPH, 19 PIN with no associated cancer, 25 PCa (including 21 with associated PIN) and 45 metastatic sections from a range of distant sites. Paraffin-embedded prostate tissue sections were processed as previously described (Burger *et al*, 2002). The paraffin tissue sections were rehydrated in xylene for 5 min and rinsed in 100% ethanol followed by 70% (v/v) ethanol and PBS. Antigen retrieval within the sections was performed by covering the slides in citrate buffer for 15 min at 105°C and left to cool to room temperature. The slides were incubated in 2% (v/v) hydrogen peroxidase for 15 min to inactivate endogenous peroxidases. Slides were washed in PBS and blocked with 10% (v/v) goat serum and the primary antibody (anti-claudin-4 raised in mouse, 3 *μ*g ml^−1^; anti-AMACR raised in mouse, 3 *μ*g ml^−1^; Zymed) overnight in a humidifier chamber. Control slides were treated as above without the addition of the primary antibody. The following day, sections were washed twice in PBS for 5 min and incubated with anti-mouse IgG HRP-conjugated secondary antibody (DakoCytomation, EnVision+® System: Dako Australasia Pty Ltd, Kingsgrove, Australia) for 20 min at room temperature. The labelled secondary antibody was visualised by adding a substrate containing diaminobenzadine (Zymed), and sections were counter-stained, dehydrated and mounted.

## Results

### Identification of metastatic markers for prostate cancer

**Figure 1:**
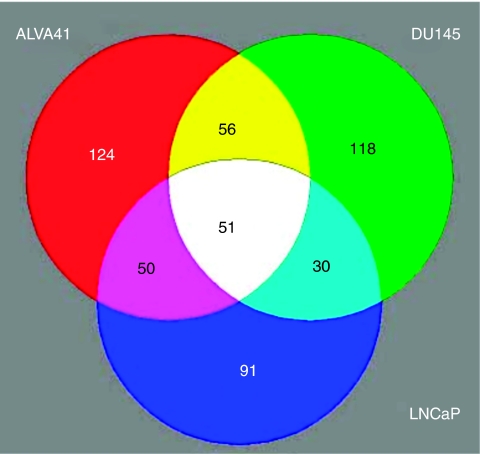
Venn diagram outlining genes upregulated in the metastatic prostate tumour cell lines. Each circle represents genes expressed approximately >twofold in a metastatic tumour cell line (ALVA41, DU145 and LNCaP) compared with the normal prostate cell line, RWPE1. The number of genes upregulated in ALVA41 (red), DU145 (green) and LNCaP (blue) was 124, 118 and 91, respectively. The overlapping regions represent genes commonly overexpressed. Genes commonly upregulated in all the three cell lines are represented in the centre white region (51 genes).

We previously identified a number of genes upregulated in PCa, which when used in combination represent useful biomarkers for detection of this disease (Landers *et al*, 2005). As an extension of this approach to relate these and additional markers of primary PCa to PCa metastases, gene expression profiling was carried out for three PCa metastatic tumour cell lines (ALVA41, DU145 and LNCaP) and compared with the normal prostate cell line, RWPE1. Reference RNA was also included by pooling total RNA from a selection of different cell types. The cell lines were indirectly labelled with Cy dyes and hybridised to an approximately 4600 gene in-house cDNA microarray chip. After an initial identification of the genes upregulated in the pooled tumour lines compared with the control cell line, RPWE1, these genes were plotted on a Venn diagram that allowed for identification of a short list of 51 genes upregulated twofold or greater, common to all three cell lines (Figure 1). A list of these genes is presented in Table 1. Potential biomarker candidate genes were selected not only on the basis of fold change but also on the basis of a number of other criteria that included twofold upregulation, published literature describing expression in prostate, PCa or other cancers, function, potential as a biomarker and whether the gene encoded a membrane protein suitable for possible use in a diagnostic test. On the basis of these criteria, we radically reduced the number of candidate genes to four, *claudin-4*, defender against cell death 1 (*DAD1*), peroxiredoxin 1 (*POX1*) and tumour protein D52 (*TPD52*), which were upregulated 3.7-, 2.1-, 3.3- and 3.2-fold, respectively (Table 1).

**Figure 2:**
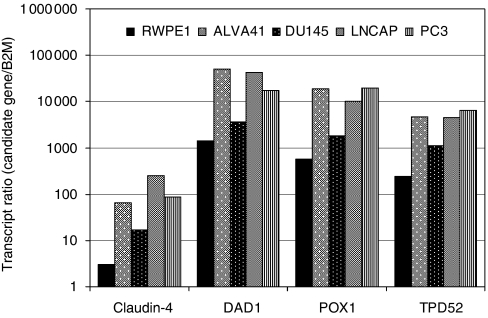
Claudin-4, DAD1, POX1 and TPD52 expression in PCa cell lines was assessed by quantitative real-time PCR. The transcript ratios (candidate gene/transcripts of *β2M*) for each gene were plotted on a logarithmic-scale graph (*x* axis). The candidate genes were examined in duplicate within each PCR run, in at least two independent experiments.

**Figure 3:**
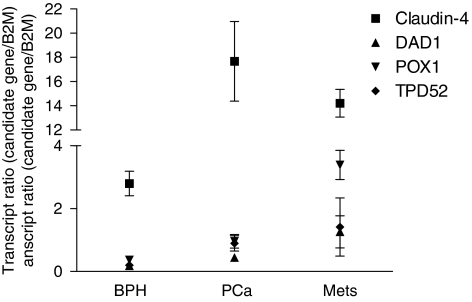
*Claudin-4, DAD1, POX1* and *TPD52* expression in BPH, PCa and metastatic tissue samples was assessed using quantitative real-time RT–PCR. The transcript ratios (candidate gene transcripts/*β2M* transcripts; *y* axis) were plotted against patient samples (BPH, PCa and metastasis; *x* axis). The expression of each candidate gene was determined in a minimum of 18 BPH, 17 PCa and 5 metastatic tissue samples. Error bars represent s.e. (standard error).

Confirmation of overexpression of these four genes was then undertaken using real-time RT–PCR. When upregulation of *claudin-4*, *DAD1*, *POX1* and *TPD52* was averaged across the three PCa cell lines compared with RWPE1, values of 35-, 20-, 23- and 17-fold respectively, were observed (Figure 2), confirming data from the cDNA microarray. Quantitative PCR was also employed to compare the expression of these genes in BPH, primary tumours and metastatic lesions. *Claudin-4* was significantly (*P*=0.00001) upregulated by sixfold in PCa specimens (mean=17.7) compared with BPH (mean=2.8; Figure 3 and Table 2). Similarly, *claudin-4* was significantly (*P*=0.00001) upregulated by fivefold in PCa metastases (mean=14.2) compared with BPH. No significant difference was observed between primary PCa and PCa metastases. *DAD1* was significantly (*P*=0.005) upregulated twofold in PCa (mean=0.44) compared with BPH (mean=0.18; Figure 3 and Table 2). The sevenfold difference between PCa metastases (mean=1.26) and BPH was significant (*P*=0.0001) as well as the threefold difference between PCa and PCa metastases (*P*=0.003). *POX1* was expressed significantly (*P*=0.012) threefold higher in PCa (mean=0.96) compared with BPH (mean=0.36) and ninefold (*P*=0.001) higher in BPH compared with PCa metastases (mean=3.4) and fourfold (*P*=0.003) higher in PCa metastasis compared with PCa (Figure 3; Table 2). *TPD52* was significantly (*P*=0.007) overexpressed in PCa (mean=0.89) compared with BPH (mean=0.20) by fivefold. The sevenfold difference between PCa metastases (mean=1.41) and BPH was significant (*P*=0.008) but the twofold difference between PCa and PCa metastases was not significant (*P*=0.066). Although the difference in transcript ratio between BPH and PCa was significant for all four biomarkers, claudin-4 was selected for further studies because of its high differential level of expression overall.

### Expression and localisation of claudin-4 in prostate cancer cell lines

**Figure 4:**
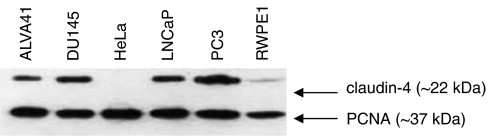
Detection of claudin-4 in cell lines using western blot analysis. Cell lysates from the metastatic prostate cell lines (ALVA41, DU145, LNCaP and PC3), a normal prostate cell line (RWPE1) and a negative-control cell line (HeLa) were run on a 15% SDS-PAGE under reducing conditions and probed with an anti-claudin-4 antibody. High levels of claudin-4 were visible in the prostate tumour cell lines with a low level detected in RWPE1. No claudin-4 was detected in the negative control cell line, HeLa. Detection of the proliferating cell nuclear antigen (PCNA) served as a loading control.

Immunoblotting was used to verify whether the increase in *claudin-4* transcript was also observed at the protein level. Claudin-4 was present in all the metastatic prostate tumour cell lines and decreased in the order of expression, PC3, DU145, LNCaP and ALVA41 (Figure 4). Low levels of claudin-4 protein were also detected in RWPE1, but, as expected, no protein was detected in HeLa cells. The level of proliferating cell nuclear antigen (*β*-actin ∼42 kDa) served as a loading control (Figure 4).

**Figure 5:**
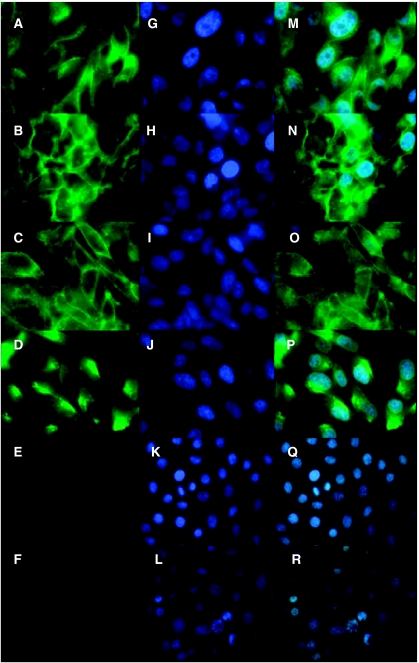
Immunofluorescent localisation of claudin-4 in cell lines. Claudin-4 localisation was examined in the adherent cell lines ALVA41 (**A**), DU145 (**B**), LNCaP (**C**), PC3 (**D**), RWPE1 (**E**) and HeLa (**F**). Claudin-4 was detected with an anti-claudin-4 antibody and visualised with an anti-mouse IgG antibody conjugated to FITC (**A**–**F**). Nuclei of cells were counterstained with DAPI (**G**–**L**). Images were merged (**M**–**R**) to determine correct localisation. The metastatic prostate tumour cell lines ALVA41 (**M**), DU145 (**N**), LNCaP (**O**) and PC3 (**P**) revealed claudin-4 to be localised to the cell membrane between cell-to-cell contact sites. The normal prostate cell line RPWE1 (**Q**) and the negative control cell line HeLa (**R**) did not reveal any claudin-4 localisation.

Immunofluorescence was used to determine the localisation of claudin-4 protein in the different cell lines (Figure 5A–F). The nuclei of cells were stained with DAPI (Figure 5G–L) and the images merged (Figure 5M–R). Claudin-4 staining was localised predominantly in the cell membrane, with diffuse staining in the cytoplasm of the metastatic prostate cell lines ALVA41, DU145, LNCaP and PC3 (Figure 5M–P). Some membranous staining was observed in ALVA41, DU145 and LNCaP cells (Figure 5M–O). However, PC3 cells had a localised region of the membrane with intense claudin-4 staining (Figure 5P). No claudin-4 staining was evident in RWPE1 or the negative control HeLa cell line above background (Figure 5Q–R).

### Expression of claudin-4 in prostate tissue

**Figure 6:**
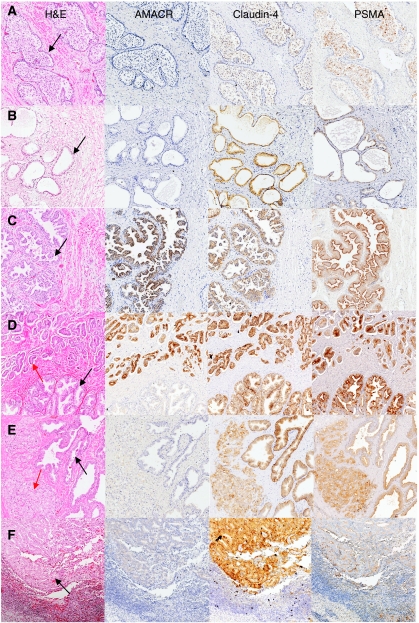
Immunohistochemical staining of AMACR, claudin-4 and PSMA representative of that observed in a range of prostate conditions. Adjacent sections were stained with haematoxylin and eosin (H and E), AMACR, claudin-4 and PSMA for each prostate tissue examined. Tissue sections consist of a normal (**A**), BPH (**B**), HG-PIN without invasive carcinoma (**C**), HG-PIN with invasive carcinoma (**D** and **E**) and metastatic carcinoma (lymph node; **F**). (**A** and **B**) Arrows indicate benign glands; (**C**) arrow indicates HG-PIN; (**D**) black arrow indicates HG-PIN and red arrow indicates PCa (Gleason 3+4); (**E**) black arrow indicates benign epithelial cells and red arrow indicates PCa (Gleason 5+5); (**F**) arrow indicates cancerous cells present at a metastatic site.

As claudin-4 was expressed at variable amounts in the prostate cell lines, it was important to determine whether upregulation and overexpression were also present in PCa primary and secondary tissues. Intensity of claudin-4 expression was classified as negative (−), light (+), moderate (++), strong (+++) or intense positive (++++). The percentage of cells stained were categorised as either < 25, 25–50% or >50% of cells within the section. In addition, staining was compared with that of PSMA and AMACR (Jiang *et al*, 2001; Rubin *et al*, 2002). Immunohistochemical analysis was undertaken on a range of prostatic sections including, normal prostate, BPH, HG-PIN, PCa and metastases. Benign epithelium in which claudin-4 and PSMA did stain positively was localised to the cell membrane of luminal cells. Membranous staining for claudin-4 and PSMA was also observed in luminal cells of HG-PIN and in carcinoma cells. AMACR was localised to the cytoplasm of luminal cells of benign epithelium or HG-PIN and to the cytoplasm of carcinoma cells. Basal epithelium and the surrounding stromal cells did not stain positively for claudin-4, AMACR or PSMA. These findings are illustrated in Figure 6. AMACR staining was localised to the cytoplasm of cells. Claudin-4 staining was localised predominantly to the membrane with weaker cytoplasmic staining, which seemed to be variable. Similarly, PSMA was localised predominantly to the membrane.

### Normal prostate sections

In the 29 normal prostate sections collected, claudin-4 was localised to the cell membrane of luminal cells at a moderate intensity in 24% (7 out of 29), at a low intensity in 66% (19 out of 29) and was absent in 10% (3 out of 29) of the sections (Figure 6A; Table 3). Approximately 25–50% of the cells stained positively in most sections. There was no correlation with level of claudin-4 expression in the prostate and age. In the normal prostate sections, AMACR staining was weak in 20% (3 out of 15) of sections and absent in 80% (12 out of 15). Prostate-specific membrane antigen was present at moderate levels of expression in 14% (2 out of 14), at low levels in 79% (11 out of 14) and was absent in 7% (1 out of 14) sections.

### Benign prostatic hyperplasia

From 19 BPH sections, consisting of nodules of hyperplastic glands and intervening stroma or stromal nodules (Foster, 2000), claudin-4 staining was strong in 10.5% (2 out of 19) of cases and moderate in 63.2% (12 out of 19) (Figure 6B; Table 2). The remaining sections had either low (21%; 4 out of 19) or no (5.26%; 1 out of 19) claudin-4 staining. Staining was localised to the membrane and in some cases to the cytoplasm of acinar cells. Weak AMACR staining was detected in 14% (2 out of 14), and the remaining 86% (12 out of 14) did not reveal any staining. Prostate-specific membrane antigen was expressed moderately in 31% (4 out of 13) and weakly in 54% (7 out of 13; Figure 6: Table 3). The remaining sections (15%; 2 out of 13) did not have any PSMA present.

### High-grade prostatic intraepithelial neoplasia

Both focal and extensive *HG-PIN*, characterised by progressive basal layer disruption, loss of markers of secretory differentiation, nuclear and nucleolar abnormalities, increasing proliferative potential, microvessel density, variation in DNA content and allelic loss (Bostwick *et al*, 2004), were studied. Initially, 18 sections with HG-PIN without invasive carcinoma were examined for claudin-4 expression. From the 18 HG-PIN sections obtained, 72% (13 out of 18) were of a moderate intensity for claudin-4 expression (Figure 6C; Table 3). Within these sections, >50% of the cells were positive for claudin-4, with staining localised to the membrane of HG-PIN cells. The remaining 11% (2 out of 18) of sections were strong or 17% (3 out of 18) low. AMACR staining was high in 9% (1 out of 11), moderate in 9% (1 out of 11), low in 36% (4 out of 11) and absent in 46% (5 out of 11) of HG-PIN in the absence of invasive carcinoma. Prostate-specific membrane antigen was present at a high level in 9% (1 out of 11), at a low level in 55% (6 out of 11) and absent in 36% (4 out of 11) of sections.

### HG-PIN in the presence of PCa

As PCa is commonly associated with the presence of HG-PIN (Bostwick *et al*, 2000), PCa sections were collected from 25 individuals, with 21 of the sections containing HG-PIN and PCa. Strong staining for claudin-4 was present (71%; 15 out of 21), with moderate staining in 28.5% (6 out of 21) or >25% of cells (Figure 6D; Table 3). On the other hand, the majority of the invasive tumour cells studied exhibited moderate staining (64%; 16 out of 25) for claudin-4, whereas 24% (6 out of 25) were strong and 8% (2 out of 25) low. Interestingly, lower grade (Gleason grade 3) tumours had higher staining for claudin-4 compared with higher grade (Gleason grade 5) tumours (Figure 6E; Table 3). Furthermore, benign glands in the PCa sections were moderate (56%; 14 out of 25) to high (44%; 11 out of 25) for claudin-4 staining and, in some cases, higher than that found in invasive carcinoma cells.

High-grade prostatic intraepithelial neoplasia cells found within PCa sections demonstrated low (32%; 6 out of 19) to moderate (21%; 4 out of 19) levels of expression for AMACR, with a small percentage staining high (10%; 2 out of 19). The remaining 37% (7 out of 19) did not have AMACR present. In invasive carcinoma cells, AMACR staining was intense in 5% (1 out of 21), high in 33% (7 out of 21), moderate in 24% (5 out of 21), low in 24% (5 out of 21) and absent in 14% (3 out of 21) of cases. Benign epithelial cells in those sections showed low staining for AMACR (5%; 1 out of 21), whereas the remaining (95%; 20 out of 21) sections did not show AMACR. Prostate-specific membrane antigen staining of HG-PIN cells in the presence of invasive carcinoma was high in 17% (3 out of 18) of sections, moderate in 44% (8 out of 18) and low in 39% (7 out of 18). In contrast, PSMA was high in 38% (8 out of 21) of sections containing invasive carcinoma cells, moderate in 38% (8 out of 21) and low in 24% (5 out of 21). The benign cells in these sections were high for PSMA in 33% (7 out of 21) of PCa sections and low in 52% (11 out of 21) of sections. The remaining 14% (3 out of 21) of sections did not have any PSMA present (Table 3).

### PCa metastatic lesions

Although lymph node and bone metastases predominate in patients with advanced PCa (Bubendorf *et al*, 2000), lesions from a wide range of secondary sites were studied (45 sections in total). Specimens from both androgen-intact and androgen-suppressed patients were isolated from a range of tissues including lymph nodes (18 out of 45), bone (8 out of 45), liver (1 out of 45), lung (1 out of 45), penile (2 out of 45), seminal vesicles (2 out of 45) and biopsies of pelvic retroperitoneal tumours (13 out of 45). From the 45 tumour metastases sections examined, 36% (16 out of 45) had strong, 29% (13 out of 45) had moderate, 20% (9 out of 45) had low and 7% (3 out of 45) had negative staining for claudin-4. It was also observed that a small percentage (9%; 4 out of 45) of metastases showed intense staining for claudin-4. Furthermore, in 47% of sections of metastases, >50–100% of carcinoma cells stained positively for claudin-4 (Figure 6F; Table 3). Those sections with intense claudin-4 staining included liver, lymph node, bone and lung metastases. AMACR expression was high in 16% (6 out of 38) of sections and moderate in 92% (11 out of 38). Twenty-six per cent (10 out of 38) of the sections had low staining for AMACR, whereas the remaining (29%; 11 out of 38) of sections had no AMACR staining. Prostate-specific membrane antigen staining of sections with metastases revealed high levels in 44% (7 out of 16), moderate levels in 31% (5 out of 16) and low levels in 25% (4 out of 16) of sections (Table 3).

## Discussion

In previous work, we examined the expression profiles of prostatic tissues using cDNA microarrays, which revealed *δ*-Catenin and PSMA to be significantly overexpressed in primary PCa tumours compared with BPH (Burger *et al*, 2002). In addition, we revealed that a combination of four PCa biomarkers, UDP-*N*-acetyl-*α*-D-galactosamine transferase (GalNAc-T3), PSMA, Hepsin and DD3/PCA3 represented a powerful new approach for detecting all PCa cells by molecular profiling (Landers *et al*, 2005). The present study was designed to extend this approach to encompass metastases in addition to primary PCa. Fifty-one genes were upregulated in all three of the metastatic cell lines and of these *claudin-4, DAD1, POX1* and *TPD52* were selected for detailed evaluation. From the four candidate genes, our findings indicated that *claudin-4* had the greatest potential as a PCa biomarker for both primary tumours and metastases.

Claudins are essential components of tight junction structures (Morita *et al*, 1999) and show a distinct organ-specific distribution in the body (Rahner *et al*, 2001). Claudin-4 encodes a 209 amino-acid (22 kDa) protein that contains four putative transmembrane regions (Katahira *et al*, 1997a) sharing close homology with claudin-3. Both claudin-4 and claudin-3 (to a lesser extent) function as receptors for *Clostridium perfringens* enterotoxin (CPE) (Katahira *et al*, 1997b), the virulence factor responsible for the symptoms of *C. perfringens* strain A food poisoning. By targeting intestinal epithelial cells, CPE is thought to act by forming small pores to increase membrane permeability with a subsequent loss of osmotic equilibrium resulting in cell lysis (Stark and Duncan, 1971; Matsuda and Sugimoto, 1979).

There have been previous reports to indicate that claudin-4 is upregulated in primary breast (Kominsky *et al*, 2004; Tokes *et al*, 2005), ovarian (Rangel *et al*, 2003; Agarwal *et al*, 2005; Santin *et al*, 2005), prostate (Long *et al*, 2001; Sheehan *et al*, 2007) squamous cell carcinoma (Morita *et al*, 2004) and pancreatic cancers (Michl *et al*, 2001, 2003; Nichols *et al*, 2004; Sato *et al*, 2004) but is downregulated in gastric cancer (Lee *et al*, 2005). In this study, the increasing prominence of claudin-4 compared with other candidate genes evaluated at both RNA and protein levels was confirmed with highest levels in PCa metastatic cells. Long *et al* (2001) observed claudin-4 expression in metastatic prostate tissue samples; however, they failed to observe expression in the metastatic cell lines LNCaP and PC3, attributing this inconsistency to the possibility of alterations induced with long periods of passaging (Long *et al*, 2001). Interestingly, we found that claudin-4 localisation in PC3 cells was confined to only a sector of the membrane but the reasons for this finding are unclear. One possible explanation is a loss of cellular organisation due to a defect in tight junction formation or cell polarity, features common in tumour cells (Weinstein *et al*, 1976).

Immunohistochemical staining *in situ* confirmed localisation of claudin-4 and PSMA to the membrane of luminal cells of benign epithelial glands and HG-PIN as well as PCa cells (Chang *et al*, 1999), with increasing expression for claudin-4 from benign, through premaligant to malignant, being most evident in metastatic PCas. In the few cases in which AMACR was present in benign glands or HG-PIN, it was localised to the cytoplasm of luminal cells (Hameed and Humphrey, 2005) with a similar localisation in PCa cells. Normal prostate sections exhibited a low level of staining for claudin-4 and PSMA, but the majority was negative for AMACR. Benign prostatic hyperplasia glands stained primarily with a moderate intensity for claudin-4 compared with PSMA and AMACR, which stained at low intensity and negatively, respectively.

High-grade prostatic intraepithelial neoplasia, previously considered to be an invariable precursor lesion of PCa and commonly found in association with PCa, does not always proceed to invasive carcinoma and, when it does, this may develop decades later (Montironi *et al*, 2002). In considering this relationship, sections containing HG-PIN without associated invasive carcinoma were of moderate intensity for claudin-4 and of low intensity for PSMA and AMACR. Cells containing HG-PIN in association with invasive carcinoma exhibited strong levels of claudin-4 staining. In contrast, AMACR levels were low and PSMA levels moderate. This observation that claudin-4 expression is increased in HG-PIN in the presence of PCa may indicate that claudin-4 plays a role in the early events of PCa development.

Although a moderate level of staining for claudin-4 was evident in the majority of invasive PCa cells, a closer examination of tumour sections revealed that claudin-4 expression tended to be higher in lower grade carcinomas compared with those of higher grades (Gleason 5) (data not shown). Furthermore, epithelial cells in surrounding benign glands within the PCa sections also had moderate to strong claudin-4 staining. As cellular organisation is lost in cancer, not uncommonly a reduction in tight junction function is observable, consistent with changes in cellular polarity associated with increased cellular mobility (Weinstein *et al*, 1976).

Prostate cancer metastases examined by immunohistochemistry were from a range of secondary sites. Most of the metastatic tumours stained positively for claudin-4, with the majority classified as strong or intense and only a minority registering moderate expression. No difference was evident in specimens from androgen-suppressed and nonandrogen-suppressed patients (data not shown). Furthermore, there did not appear to be a correlation with type of metastatic site and the intensity of claudin-4 expression: those sections with the strongest claudin-4 staining were from liver, lymph node, bone and lung. Although decreased polarity and differentiation are regarded as important for the metastatic phenotype, to enable individual cells to leave the primary site and enter the circulation to reach distant sites (Martin and Jiang, 2001), circumstances are different in established metastases in which a reversal is required to permit growth of established secondary lesions (Yang *et al*, 2006).

Sheehan *et al* (2007) in their evaluation of 141 RP specimens reported an increase in claudin-4 expression in advanced-stage tumours compared with adjacent benign glands and concluded that claudin-4 expression persisted in PCa and correlated with an adverse prognosis. In contrast, our study of both primary and secondary lesions examined 182 sections from patients with a range of prostate conditions to provide a better understanding of the role of claudin-4 in the prostate and in tumour progression: our findings are consistent with current concepts of the carcinogenesis/invasion process. A further difference to the report from Sheehan *et al* (2007) is that our immunohistochemical analysis of claudin-4 was supported by a comparative evaluation of AMACR and PSMA, two well-established PCa markers.

In a small study of pancreatic cancer, Michl *et al* (2003) concluded that claudin-4 expression tended to be stronger in well-differentiated tumours compared with poorly differentiated tumours, which correlates with the expression pattern observed within PCa primary lesions in this study. Also, in primary pancreatic cancer sections, Nichols *et al* (2004) reported that immunohistochemical expression of claudin-4 was present in pancreatic intraepithelial neoplasia cells and in all of the metastatic cancers examined (Nichols *et al*, 2004) reflecting our observation that claudin-4 expression was present in all HG-PIN sections associated with PCa.

It is not known what regulates claudin-4 expression; however, studies in pancreatic cancer have implicated the TGF-*β* pathway (Michl *et al*, 2003). TGF-*β* is known as a potent mediator of tumour progression by inducing cell spreading, migration, angiogenesis and tumour cell invasion (Wikstrom *et al*, 1998; Massague *et al*, 2000). A study into the regulation of claudin-4 by TGF-*β* revealed that this cytokine downregulated claudin-4 expression within pancreatic cancer, which may be the mechanism by which TGF-*β* promotes tumour invasion (Michl *et al*, 2003). Furthermore, inhibition of Ras signalling by dominant-negative Ras and specific inhibitors of downstream effectors, mitogen-activated protein/extracellular signal-regulated kinase kinase and phosphatidylinositol 3′-kinase, has been suggested to decrease claudin-4 expression (Michl *et al*, 2003). More recently, overexpression of claudin-4 in ovarian cancer was found to be partly regulated by a small region in the claudin-4 promoter-containing Sp1 sites (Honda *et al*, 2006). The claudin-4 promoter is also controlled by epigenetic modifications (Boireau *et al*, 2007). It was revealed that cells overexpressing claudin-4 exhibited low DNA methylation and high histone H3 acetylation of the critical claudin-4 promoter region with the converse observed for cells expressing low levels of claudin-4 (Boireau *et al*, 2007). Studies are currently underway in our laboratory to determine if this mechanism is also responsible for the regulation of claudin-4 in the prostate.

The results obtained within this study have revealed a distinct and progressing pattern of claudin-4 expression within the prostate with noncancerous pathological changes as well as in PCa and PCa metastases by RT–PCR and immunohistochemistry of both primary and secondary tumours. In addition, elevated levels of claudin-4 were observed in suspected premalignant and malignant lesions.

Many genes and proteins have been proposed as useful markers for diagnosis, imaging and therapeutic targeting in PCa. In diagnosis, the large proportion of those nominated was related to an aggressive phenotype with relatively few identified as potential indicators of low risk PCa. With burgeoning numbers of this category of patients being identified as a consequence of PSA screening, there is a pressing need for low-risk disease markers, particularly for those men considering close observation (Smith and Catalona, 1994; Hoedemaeker *et al*, 2000). However, before claudin-4 can be considered for any role in this regard despite its proclivity to be overexpressed preferentially in lower Gleason score tumours, further evaluation is essential. Similarly, its potential as a target for imaging or therapy must remain speculative until further definitive research is undertaken.

## Figures and Tables

**Table 1:**
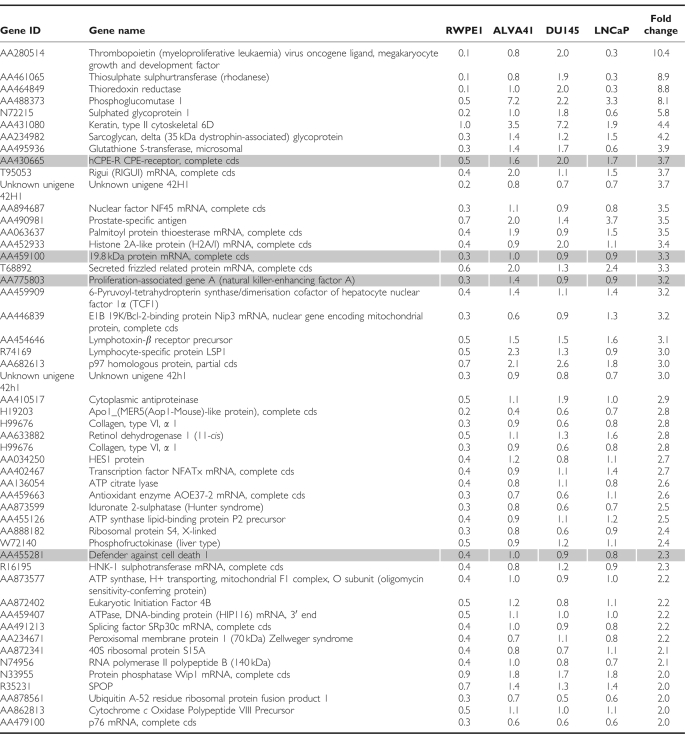
Fifty-one genes upregulated in the metastatic prostate tumour cell lines, ALVA41, DU145 and LNCaP compared with the normal prostate cell line, RWPE1

**Table 2:**
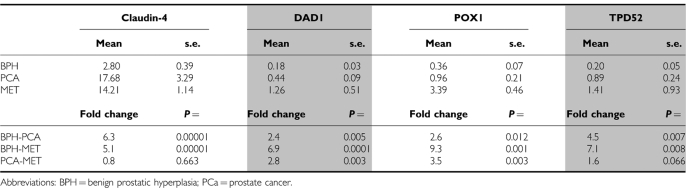
Summary of real-time RT-PCR results

**Table 3:** Immunohistochemical evaluation of AMACR, PSMA and claudin-4 expression in prostate tissue sections

	**Percentage and intensity of cells staining**								
	**<25%**	**25–50%**	**>50%**	**<25%**	**25–50%**	**>50%**	**<25%**	**25–50%**	**>50%**
*Normal prostate sections*	AMACR (*n*=15)			PSMA (*n*=14)			Claudin-4 (*n*=29)		
Nil	12			1			3		
Low	3			3	6	2	3	12	4
Moderate				1	1			5	2
Strong									
Intense									
									
*BPH*	AMACR (*n*=14)			PSMA (*n*=13)			Claudin-4 (*n*=19)		
Nil	12			2			1		
Low	1	1		1	2	4		1	3
Moderate				1	2	1	1	6	5
Strong									2
Intense									
									
*HG-PIN (with no foci of PCa)*	AMACR (*n*=11)			PSMA (*n*=11)			Claudin-4 (*n*=18)		
Nil	5			4					
Low	3		1		6		1		2
Moderate	1						1		12
Strong		1			1		1	1	
Intense									
									
*PCa*	AMACR (*n*=21)			PSMA (*n*=21)			Claudin-4 (*n*=25)		
Nil	3								
Low	3	2		1	2	2		1	1
Moderate	1	2	2		3	5	3	9	4
Strong		1	6	1	1	6	3	2	1
Intense			1					1	
									
*HG-PIN (with foci of PCa)*	AMACR (*n*=19)			PSMA (*n*=18)			Claudin-4 (*n*=21)		
Nil	7								
Low	4	2		4	2	1			
Moderate	2		2		3	5		2	4
Strong	1		1			3	1	8	6
Intense									
									
*Benign glands (adjacent to PCa)*	AMACR (*n*=21)			PSMA (*n*=21)			Claudin-4 (*n*=25)		
Nil	20			3					
Low	1			5	3	3			
Moderate				2	4	1		7	7
Strong							2	6	3
Intense									
									
*Metastatic sites*	AMACR (*n*=18)			PSMA (*n*=16)			Claudin-4 (*n*=45)		
Nil	11						3		
Low	5	4	1	3	1		9		
Moderate	6	2	3		1	4	2	6	5
Strong			6	4	2	1		3	13
Intense								1	3

Abbreviations: AMACR=*α*-methylacyl-CoA racemase; BPH=benign prostatic hyperplasia; HG-PIN=high-grade prostatic intraepithelial neoplasia; PCa=prostate cancer; PSMA=prostate-specific membrane antigen.

Immunohistochemical staining for AMACR, PSMA and claudin-4 was performed on a number (*n*) of prostate sections. The percentage and the intensity of cells staining within each of the sections were determined by a uropathologist.
